# Removal of a Past Varnish Treatment from a 19th-Century Belgian Wall Painting by Means of a Solvent-Loaded Double Network Hydrogel

**DOI:** 10.3390/polym13162651

**Published:** 2021-08-10

**Authors:** Ehab Al-Emam, Victoria Beltran, Steven De Meyer, Gert Nuyts, Vera Wetemans, Karolien De Wael, Joost Caen, Koen Janssens

**Affiliations:** 1 Department of Conservation, Faculty of Archaeology, Sohag University, 82524 Sohag, Egypt; 2 AXES, Faculty of Science, University of Antwerp, Groenenborgerlaan 171, 2020 Antwerp, Belgium; victoria.beltran@uantwerpen.be (V.B.); steven.demeyer@uantwerpen.be (S.D.M.); gert.nuyts@uantwerpen.be (G.N.); karolien.dewael@uantwerpen.be (K.D.W.); koen.janssens@uantwerpen.be (K.J.); 3 ARCHES, Faculty of Design Sciences, University of Antwerp, Mutsaardstraat 31, 2000 Antwerp, Belgium; wetemansvera@gmail.com (V.W.); joost.caen@uantwerpen.be (J.C.)

**Keywords:** FTIR spectroscopy, gel cleaning, optical microscopy, SEM-EDX, solvent-loaded hydrogels, deteriorated synthetic varnish, varnish removal, wall painting, XRD

## Abstract

Polymeric materials have been used by painting conservator-restorers as consolidants and/or varnishes for wall paintings. The application of these materials is carried out when confronting loose paint layers or as a protective coating. However, these materials deteriorate and cause physiochemical alterations to the treated surface. In the past, the monumental neo-gothic wall painting ‘The Last Judgment’ in the chapel of Sint-Jan Berchmanscollege in Antwerp, Belgium was treated with a synthetic polymeric material. This varnish deteriorated significantly and turned brown, obscuring the paint layers. Given also that the varnish was applied to some parts of the wall painting and did not cover the entire surface, it was necessary to remove it in order to restore the original appearance of the wall painting. Previous attempts carried out by conservator-restorers made use of traditional cleaning methods, which led to damage of the fragile paint layers. Therefore, gel cleaning was proposed as a less invasive and more controllable method for gently softening and removing the varnish. The work started by identifying the paint stratigraphy and the deteriorated varnish via optical microscopy (OM), scanning electron microscopy coupled with energy-dispersive X-ray spectroscopy (SEM-EDX), X-ray diffraction (XRD), and Fourier-transform infrared (FTIR) spectroscopy. A polyvinyl alcohol–borax/agarose (PVA–B/AG) hydrogel loaded with a number of solvents/solvent mixtures was employed in a series of tests to select the most suitable hydrogel composite. By means of the hydrogel composite loaded with 10% propylene carbonate, it was possible to safely remove the brown varnish layer. The results were verified by visual examinations (under visible light ‘VIS’ and ultraviolet light ‘UV’) as well as OM and FTIR spectroscopy.

## 1. Introduction

Natural and synthetic polymers are often applied on wall paintings during conservation-restoration treatments. Conservators-restorers apply these materials to consolidate fragile paint layers or as a protective coating layer/varnish. Natural polymers, such as casein, egg yolk, animal glue, and beeswax, have been commonly used in Europe since ancient times [1]. Since the mid-1920s, synthetic polymers replaced natural ones because they were assumed to be more durable and stable. These polymers include acrylic resins (e.g., polymethyl methacrylate, poly(n-butyl) methacrylate, and poly(isobutyl) methacrylate), cellulose derivatives (e.g., carboxymethyl cellulose, cellulose nitrate, and cellulose acetate), and vinyl acetate derivatives (e.g., polyvinyl acetate, polyvinyl alcohol, and polyvinyl acetal) [1,2,3]. Once they are applied, many of these natural and synthetic materials tend to change the physiochemical properties of the treated surface of wall paintings and become yellow or darken upon degradation [4,5].

Traditionally, varnish removal methods ranged from mechanical cleaning (using scalpels) to chemical cleaning (using free solvent/solvent mixtures or nanostructured fluids) [6,7,8,9,10] but also included noncontact cleaning methods (i.e., laser cleaning) [11,12,13]. More recently, gelling materials have been employed as improved chemical cleaning methods by confining solvents and other cleaning agents to the 3D network of the gel, leading to less infiltration of the cleaning agents into the painting surface. This cleaning method allows the deployment of a less invasive treatment, which is essential for delicate painted surfaces [14,15,16].

In the last two decades, several types of gelling materials have been widely used for removing varnish layers from painted surfaces. For instance, PVA–borax hydrogel incorporated with 1-propanol had the ability to remove deteriorated brown varnish from a 16th–17th-century oil painting [17]. A PVA–borax gel loaded with acetone was used to clean a highly oxidized shellac layer from a 15th-century egg tempera wood panel [18]. In addition, water-based PVA–borax films (in which no organic solvents were incorporated) were used to remove oxidized shellac varnish from two icons from the 19th-century. This cleaning method was enhanced using a thermal spatula in order to remove a thick layer of shellac and dirt from the surface of the artwork [19]. An organogel composed of 40% hydrolyzed polyvinyl acetate crosslinked with benzene-1,4-diboronic acid in 95:5 ethanol/water was able to eliminate varnish consisting of a mixture of shellac, drying oils, and pigment [20]. Recently, a PVA twin-chain gel loaded with a nanostructured fluid allowed the safe removal of a polyvinyl acetate varnish and wax layer from an oil painting by Pablo Picasso [21].

Moskalik-Detalle et al. [22] removed old varnishes, glazes, and overpaint layers from a 19th century wall painting at Saint Sulpice, Paris. The cleaning action was executed by brushing velvesil gel mixed with hexamethyldisiloxane (D2) and benzyl alcohol; the mixture was cleared by a soft absorbent paper and rinsed with D2-moistened cotton pads. The treatment also involved prewetting the area to be cleaned with octamethylcyclotetrasiloxane (D4) to limit the deep penetration of the cleaning liquids in the paint layers. PVA–borax solvent-gels were successfully employed for removing varnish layers from 19th century icons. This cleaning method provided an improvement in the treatment over the traditional methods (i.e., scalpel and free solvents) which were risky and harmful to the paint layers [23]. In another case study, the mural paintings of Adolphe Roger at the church of Notre Dame de Lorette, Paris, which was coated with two superimposed varnishes in two different conservation campaigns, were treated with an emulsion composed of 10% benzyl alcohol and 2% xanthan gum (buffered to a pH of 8) to remove the varnish layers. The emulsion was applied using a brush for about 2 min and cleared with water (buffered to a pH of 7) [24]. In all the abovementioned cases, the use of gels provided a safe way to remove varnish and deposit layers from painted surfaces that were difficult to remove via traditional cleaning techniques.

In the present paper, we describe a challenging case study in which a deteriorated brown synthetic varnish layer needed to be removed from a 19th-century Belgian wall painting. Numerous attempts were carried out to remove the varnish using traditional cleaning methods, such as mechanical (i.e., involving scalpels and hot air) and chemical (i.e., using cotton swabbing and poultices) cleaning. All the tested cleaning methods failed to remove the varnish or gave rise to damage/detachment of the paint layers. A description of these cleaning tests is provided in the Supplementary Materials, Section S1. For this reason, we evaluated the application of double network hydrogels as a less invasive and controllable cleaning tool. In this regard, we studied the stratigraphy of the wall painting using optical microscopy (OM), scanning electron microscopy coupled with energy-dispersive X-ray spectroscopy (SEM-EDX), and X-ray diffraction (XRD), in addition to identifying the varnish layer and the binding medium by Fourier-transform infrared (FTIR) spectroscopy. Previously, this polyvinyl alcohol–borax/agarose (PVA–B/AG) double-network hydrogel was successfully employed to remove a deteriorated consolidant and soot layers from the wall paintings of the temple of Seti I, Abydos, Egypt; the hydrogel, loaded with the proper cleaning system, showed good results for safely removing the undesired deposits without causing damage to the paint layers [25,26]. For the present case of the 19th century wall painting, the hydrogel was loaded with several solvents/solvent mixtures with the ability to swell the varnish; the various loaded hydrogels were tested on small regions of the wall painting. The results were evaluated in situ by means of visual examinations under visible light and UV light as well as by ATR–FTIR spectroscopy. In the next phase, the hydrogel composite that yielded the best cleaning results was employed to safely remove the varnish layer from the wall painting on a larger scale. Additionally, an analysis of paint microsamples by µ-FTIR spectroscopy allowed us to monitor whether the cleaning methods degraded the binding medium or caused diffusion of the varnish into the surface of the wall painting, ensuring the preservation of the painting after the treatment and demonstrating the efficiency of the methodology applied.

### Historical Context

The wall painting is located in the Sint-Jan Berchmanscollege, Antwerp, Belgium. The school was named after the saint Jan Berchmans (Diest, Belgium, 1599–Rome, Italy 1632), a scholastic of the Society of Jesus [27,28].

The neo-Gothic Sint-Jan Berchmanscollege was designed by the architect Edmond Leclef and was built in 1891. In 1898, the wall painting, depicting the Last Judgment, was executed by the Belgian artist Ernst Wante (1872–1960) [29]. Wante studied at the Royal Academy and the Higher Institute of Fine Arts in Antwerp [30,31]. Later, as one of the teachers at the Antwerp Academy, he painted many landscapes and more than four hundred portraits; however, the artist is primarily known for his famous religious frescoes [32], such as the Last Judgment discussed here.

**Figure 1:**
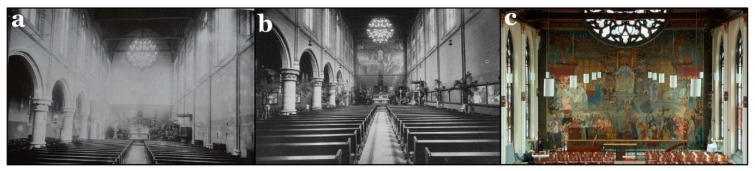
(**a**) A black and white photograph of the chapel in 1897 in the Sint-Jan Berchmanscollege, when the wall painting was not yet executed. (**b**) The chapel in the 1930s, after Ernst Wante created the wall painting. The chapel was not yet divided into two floors at that time [33]. (**c**) A photograph of the wall painting in 2017 showing its state before conservation. The wall painting is now found on the top floor of the chapel.

Originally, the wall painting occupied the top part of the eastern chapel wall, as can be seen in Figure 1a,b. Later, the chapel was divided into two levels. The lower level is now a gymnastics hall, while the upper one, where the wall painting is located, is used for celebrations, lectures, and music lessons (Figure 1c). Consistent with the surrounding architecture, Wante executed the wall painting in a neo-Gothic style.

## 2. Materials and Methods

### 2.1. Materials

Acetone (*AC*) (>95%, technical), ethanol (*EtOH*) (96%, technical), polyvinyl alcohol (*PVA*) (98.0–98.8% hydrolyzed, M.W. 146,000–186,000), and toluene (*Tol*) (99.5%, ACS reagent) were purchased from Acros Organics (Geel, Belgium). Agarose (*AG*) (molecular biology grade, low EEO/multipurpose) and ethyl acetate (*EA*) (>99%, laboratory reagent grade) were acquired from Fisher scientific (Merelbeke, Belgium). Di-sodium tetraborate decahydrate (*borax*) (assay ‘acidimetric’ 99.5%, ACS, ISO reagent) was supplied by Merck (Overijse, Belgium). Methyl ethyl ketone (*MEK*) (≥99%, technical) was obtained from VWR PROLABO (Leuven, Belgium). Propylene carbonate (*PC*) (99%) was purchased from Alfa Aesar (Kandel, Germany). White spirit (*WS) (petroleum benzine 180-210, extra pure*) was acquired from Carl Roth (Karlsruhe, Germany). 1-Pentanol (*1-PeOH*) (> 99.0%, M.W. 88.15) was supplied by TCI (Zwijndrecht, Belgium). All materials were used as received.

### 2.2. Instrumentations

#### 2.2.1. Optical Microscopy (OM)

**Figure 2:**
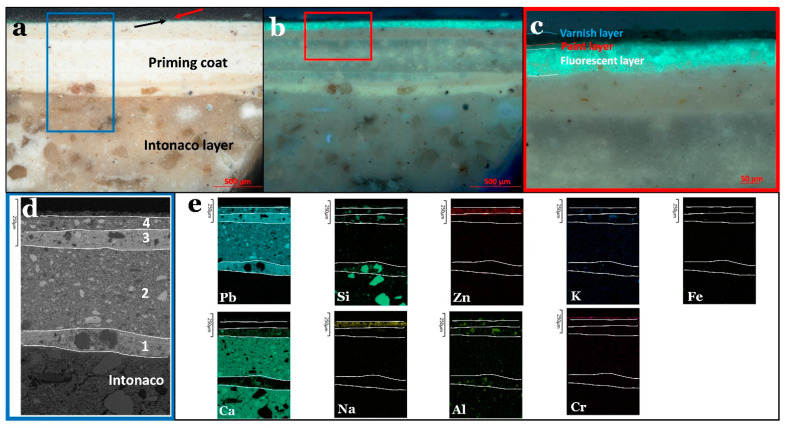
(**a**) An optical microscopy image showing the intonaco, priming, paint layer (black arrow), and varnish layer (red arrow). (**b**) The same view under UV light. (**c**) A magnified field of the red rectangular present in ‘b’. The image illustrates the fluorescent layer belonging to the priming coat in addition to the paint and the varnish layers. (**d**) A backscattered SEM image (of the area in the blue rectangle indicated in ‘a’) revealing the intonaco layer and the superimposed four layers of the priming coat. (**e**) Elemental maps acquired by SEM–EDX of the same field are presented in ‘d’.

For the examination of the cross-sections of the paint samples, a Nikon ECLIPSE LV100ND microscope attached to a CoolLED pE-4000 UV light source and equipped with a Nikon DS-Fi3 camera (Nikon, Leuven, Belgium) was used (Figure 2 and Supplementary Figure S2). An Olympus Digital Microscope DSX510 (3D microscope) allowed the verification of the efficiency of the varnish removal by the selected hydrogel composite (Olympus, Hamburg, Germany) (Figure 10a,b).

#### 2.2.2. SEM-EDX

The paint cross-sections were examined with a field emission gun–environmental scanning electron microscope (FEG–ESEM) equipped with an energy dispersive X-Ray (EDX) detector (FEI Quanta 250, Hillsboro, OR, USA; at AXES and EMAT research groups, University of Antwerp), using an accelerating voltage of 20kV, a take-off angle of 30°, a working distance of 10 mm, and a sample chamber pressure of 10^−4^ Pa. Imaging was performed using secondary electrons (SE) and back-scattered electrons (BSE). EDX maps were acquired using a beam current of ~0.5 nA.

The map presented in Figure 2 has a resolution of 1024 × 704 pixels and a size of 1.16 mm by 0.799 mm; a live time of 0.55 ms per pixel was used during the acquisition. The map shown in Supplementary Figure S2 has a resolution of 2048 × 1408 pixels and a size of 1.9 mm by 1.31 mm; this corresponds to a live time of 0.3 ms per pixel.

#### 2.2.3. XRD

A laboratory MA-XRPD scanner was used to analyze cross-sections of the paint samples to acquire information regarding the crystalline compounds used in the different layers. For this reason, a monochromatic Cu K_α_ (8.04 keV) X-ray source was employed to scan the samples in reflection mode. A primary beam impingement angle of 10° relative to the sample’s surface was chosen, leading to a beam with an elliptical footprint of approximately 1 × 0.2 mm^2^ (horizontal × vertical). The X-ray source (IμS-Cu^HB^, Incoatec GmbH, Geesthacht, Germany) generates a photon flux of 2.9 × 10^8^ photons s^−1^ and has a focal diameter of (142 ± 2) µm, a focal distance of (20 ± 1) cm, and a divergence of 2.4 ± 0.1 mrad. The samples were placed at a distance of 20 cm from the X-ray source. A PILATUS 200K (DECTRIS, Baden-Daettwil, Switzerland) detector was oriented at an angle of 40° with respect to the sample’s surface. In order to identify the compounds present throughout the stratigraphy, a line scan consisting of multiple points was performed with an exposure time of 10 s pt^−1^.

A set of three motor stages (30 cm × 30 cm × 10 cm; Newport Corp., Irvine, CA, USA) were responsible for the movements during the scanning procedure. The X-ray source and PILATUS 200K detector were placed on a motorized platform capable of moving the setup in the X, Y, and Z directions relative to the sample.

#### 2.2.4. FTIR Spectroscopy

For the identification of the varnish and organic binding medium prior to any treatment, FTIR was used. Furthermore, it was employed in the final cleaning stage to verify varnish removal, possible varnish infiltration into the layers underneath, binding medium alterations, and hydrogel residues.

**Figure 3:**
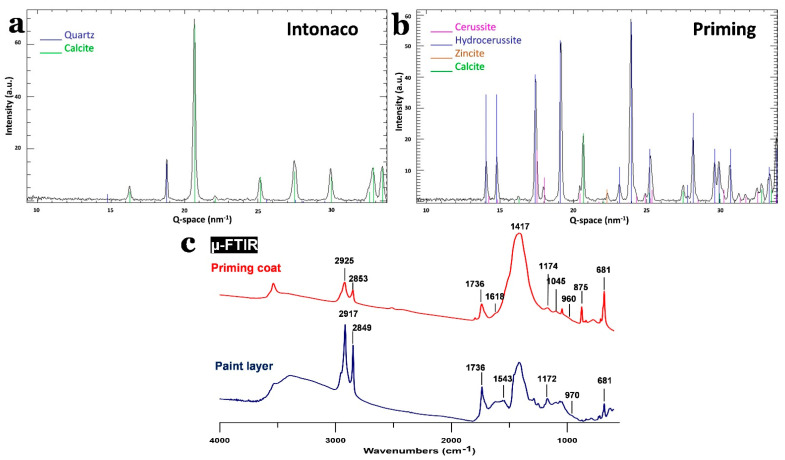
(**a**) and (**b**) XRD diffractogram of the intonaco and priming coat, respectively. (**c**) µ-FTIR spectra of the painting and priming coat.

FTIR measurements were performed using a LUMOS II spectrometer from Bruker (Kontich, Belgium). µ-FTIR measurements (Figure 3c, Figure 10c, and Supplementary Figure S6) were performed in transmission mode equipped with an MCT detector, with a spectral range of 4000–700 cm^−1^, a 4 cm^−1^ resolution, 128 scans, and a 100 × 100 µm^2^ spot size. FTIR measurements (Supplementary Figure S5) were performed using an ATR accessory equipped with a diamond crystal. At least three spectra per point were recorded and compared with each other to ensure the reproducibility of the data.

An Agilent 4300 Handheld FTIR spectrometer (Agilent, Santa Clara, CA, USA) (Figures 6 and 7) was utilized for the evaluation of the cleaning efficiency of the different hydrogel composites applied to the wall painting. The spectra were collected at a resolution of 4 cm^−1^ with 16 scans in the range of 4000–600 cm^−1^. The evaluation was based on the average of three point measurements for each cleaned spot.

### 2.3. Sample Preparation

Cross-sections of varnished paint samples were made from the different layers of the wall painting in order to yield more detailed information about the stratigraphical structure and the materials used by the artist in executing the wall painting. The cross-sections were prepared by embedding the samples in Epofix^®^ resin and cutting them with a Buehler IsoMet low speed (11-1180-250) saw. Subsequently, the samples were manually wet-polished on Microcut^®^ silicon carbide paper (1200 grit).

## 3. Results and Discussions

### 3.1. Materials and Structure of the Wall Painting

#### 3.1.1. Painting Stratigraphy and Technique

Visually, the wall painting support is essentially a red brick wall coated with a ground layer. This ground layer can be divided into a brownish coarse layer (*arriccio*) and a second finer one (*intonaco*). On top of this, a white priming coat was applied to receive the paint layer; see Supplementary Figure S1.

Under the optical microscope, the arriccio layer (thickness: c. 10 mm) was characterized by angular/subrounded particles with various sizes ranging from *c.* 50–300 µm. The color of these particles ranged from dark brown to light brown and yellowish. This layer also contained green and black particles. The use of organic fillers (such as straw and animal hair) is typical in wall paintings [34,35] (see Supplementary Figure S2a,b). The second layer (thickness: 3–7 mm) contained fewer sand particles, and their sizes ranged from *c.* 60 to 450 µm. It was also characterized by the presence of (organic) fillers, similar to the arriccio layer. The priming coat (thickness: 0.5–0.8 mm) was composed of several layers, which were difficult to differentiate using optical microscopy. This priming coat contains small particles (probably sand). In addition, examination under UV light permitted the identification of a fluorescent top layer underneath the paint layer; see Figure 2a–c.

The backscattered images and elemental maps collected by SEM-EDX revealed that the arriccio layer mainly contained Si and Ca, with the presence of Fe, Zn, Al, K, Cl, Mg, S, Ti, and Na (see Supplementary Figure S2c,e). The high concentrations of Si and Ca are consistent with the use of quartz and calcium carbonate. The elemental distribution of Fe, Al, K, Si, and Mg was correlated with the green particles observed under the optical microscope, and this may refer to green earth. The correlated presence of Fe and Ti in the black particles suggests the presence of ilmenite (iron and titanium oxide) (see Supplementary Figure S2a,c,e). The collected maps of the intonaco layer (Figure 2d and e, layer “intonaco”) included high proportions of Ca and Si in addition to minor proportions of Al, K, Fe, and Na. This suggests the employment of both calcium carbonate and quartz in this layer.

In the case of the priming coat (Figure 2d, layers 1, 2, 3, and 4), the backscattered electron image and the elemental maps allowed for better discrimination of the different layers than optical microscopy. It was observed that the priming coat was composed of four superimposed layers, which differ in composition and thickness. The first layer, with a thickness ranging from *c.* 70 to 190 µm, was rich in Pb, suggesting the use of lead carbonate considering the white color of the layer. In addition, Si was detected as quartz particles in addition to the presence of Al, Ca, K, Fe, and Na in minor amounts. Ca and Pb were present in high concentrations in the second layer (thickness = *c.* 220–450 µm), which may be attributed to the presence of a mixture of lead carbonate and calcium carbonate. Other elements were also detected in minor quantities, such as Si, Al, K, Na, and Fe. The third layer (thickness = 20–100 µm) had almost the same elemental distribution as the first, with major proportions of Pb along with the existence of the other elements (Si, Al, Na, Ca, K, and Fe) in minor proportions. The thickness of the fourth layer ranged from 30–120 µm and was mainly composed of Pb, Zn, and Na. In addition, Al, Si, Ca, K, and Fe were present. It is worth mentioning that this layer is the one that showed fluorescence under UV light. In addition, the fluorescence that can be seen in Figure 2c was directly correlated with the distribution of the element zinc (Figure 2e), suggesting that zinc oxide is probably responsible for this feature [36].

The collected XRD diffractogram from the arriccio layer supports the results acquired from the SEM-EDX. The sample was mainly composed of quartz (SiO_2_) and calcite (CaCO_3_) (see Supplementary Figure S2d. The intonaco layer showed abundant amounts of quartz and calcite (Figure 3a). The priming coat contained lead white (90% hydrocerussite–Pb_3_(CO_3_)_2_(OH)_2_–and 10% cerussite–PbCO_3_) in addition to the presence of calcite (Figure 3b), as demonstrated by the SEM-EDX Pb map. This coat also contained zincite (ZnO), as was suspected from the fluorescent fourth layer observed under OM.

Small fragments from an unvarnished painting surface and the top layers of the priming coat were extracted and analyzed with µ-FTIR spectroscopy (see Figure 3c). The collected spectra from the paint surface showed bands at 2917 and 2849 cm^−1^, corresponding to the presence of long CH_2_ chains (CH stretching); the peak at 1736 cm^−1^ was probably related to esters (C=O stretching). These bands can be linked to the presence of wax, drying oil, or a mixture of both compounds; the band at 1172 and the shoulder at 970 cm^−1^ agree with the presence of drying oil (vibrations of the polymeric chain) [37]. In addition, in the painting layer, there was a band at 1543 cm^−1^, which was related to the presence of carboxylates (C=O stretching), normally related to the reaction products of drying oil and inorganic pigments, which also have sharp bands at 2917 and 2849 cm^−1^ [38]. However, the sharpness and the high intensity of the bands at 2917 and 2849 cm^−1^ suggest the presence of a drying oil together with a wax [39]. The presence of wax would also explain the sticky texture of the painting surface.

Regarding the pigments, the characteristic markers of lead white (bands around 1417 and 1045 cm^−1^, vibration from carbonate moiety) [40] and calcium carbonate (bands around 1430 and 875 cm^−1^, vibrations from the carbonate moiety) [41] could be seen in the painting layer, which agrees with the results obtained from SEM-EDX and XRD.

The spectra collected from the priming coat also displayed bands at 2925 and 2853 cm^−1^, together with the bands at 1736, 1174, and 960 cm^−1^, indicating the presence of a drying oil. Nonetheless, the intensities of these bands were smaller than those in the painting layer, indicating that the proportion was lower. On the other hand, the broader bands at 2925 and 2853 cm^−1^ compared with the paint layer suggests that wax was present in lower amounts. Similar to the painting layer, a shoulder at 1618 cm^−1^ was noticed, probably linked to carboxylates. Additionally, there were also lead white and calcium carbonate bands, which agreed with the data collected using SEM-EDX and XRD.

The presence of wax in the wall painting was unexpected; a hypothesis to explain this could be that its presence was linked to the production of the painting by means of a mixture of a drying oil and wax as binding media or to a posterior conservation treatment. The presence of wax would provide protection against moisture and could produce a matt surface, in addition to promoting the drying speed of the paint layer [24,42]. Indeed, the encaustic painting (wax-based painting) technique was recreated in the 19th century in order to emulate durable wall paintings such as those from the Roman period [43]. Therefore, it is possible that the wax was used on purpose in the binding medium, mixed with a drying oil [22].

#### 3.1.2. Conservation State

**Figure 4:**
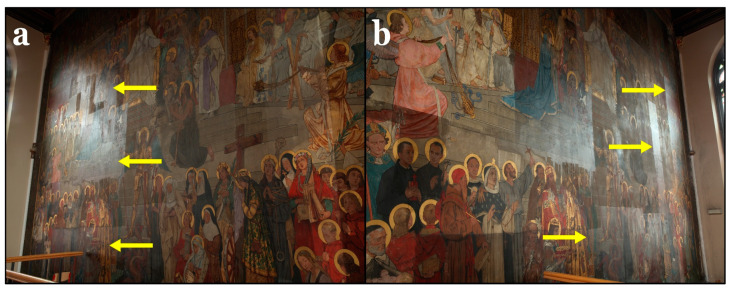
Photographs showing the glossy varnish that was not applied to the entire wall painting, as indicated by the yellow arrows. (**a**) Left side and (**b**) right side of the wall painting.

In a previous undocumented conservation campaign, the wall painting was partially coated with a varnish layer, while some areas were left uncoated. Examination of the collected samples under the microscope revealed that the thickness of this layer was in the range of 20–45 µm (Figure 2c). The varnish was probably applied to protect the paint layers and facilitate routine cleaning. Nonetheless, the varnish layer has degraded, leading to significant changes in its appearance. The most noticeable change is the severe darkening, obscuring the clarity of the painting’s figures and colors. Additionally, the varnish altered the original matt appearance of the wall painting, giving it a glossy surface (Figure 4a,b). Along with the deteriorated varnish layer, the wall painting also showed a number of other manifestations of degradation, such as paint losses, detached paint layers, hollow areas in the plaster layers, surface soiling, and lacunae.

### 3.2. Gel Cleaning Test Strategy

Peeled-off varnish drippings, created during their application by brush (Supplementary Figure S5b), were collected from three different areas of the wall painting. The analytical strategy for the gel cleaning tests included i) the identification of the varnish, ii) laboratory tests on the collected varnish samples, and iii) in situ gel cleaning tests.

The cleaning tests on the wall painting included assays with different hydrogel composites loaded with selected solvents. These tests were performed on the same colors (brown and green) on which more traditional cleaning methods were attempted (see Supplementary Figure S3). This allowed for a better evaluation of the gel cleaning compared with traditional methods. The results were evaluated using visual examinations and handheld ATR–FTIR spectroscopy in order to identify the hydrogel composite that gave the best cleaning results.

### 3.3. Varnish Identification

The collected varnish films were analyzed by ATR–FTIR spectroscopy [44,45]. All the analyzed films showed the same spectra, indicating that only one varnish type was used in the past conservation treatment.

The ATR–FTIR spectra of the varnish (see Supplementary Figure S5a) agree with the presence of a terpolymer emulsion composed of poly(*n*-butyl acrylate-methyl methacrylate/styrene). This could be seen in the spectra by the presence of its characteristic bands [46,47]. For more details regarding the characteristic bands, see Supplementary Materials, Section S2.

Since the second half of the twentieth century, acrylic emulsions were frequently used as fixatives/consolidants for wall paintings along with other acrylic polymers [1,48,49]. As mentioned earlier, these materials are susceptible to physiochemical alterations such as darkening/yellowing, as was evident from the appearance of the investigated wall painting (see Supplementary Figure S5b).

### 3.4. Laboratory Tests

#### 3.4.1. Free Solvents Solubility/Swelling Tests on the Collected Varnish Fragments

Solubility tests were conducted on the peeled-off varnish films to identify solvents capable of dissolving/swelling this layer. The solvents and mixtures chosen were ethyl acetate (EA), propylene carbonate (PC), methyl ethyl ketone (MEK), ethanol (EtOH), white spirit (WS), toluene (Tol), EA/PC (50/50%), and MEK/1-PeOH (50%/50%) since they cover the area occupied by most of the acrylic polymers on the Teas diagram, according to [2] (see Supplementary Materials: Figure S4).

Fragments of the varnish were immersed in each solvent/solvent mixture for 5 min. A proper swelling effect on the varnishes allows the separation of the varnish from the paint layer so that it can be removed with minimal mechanical effort [50]. None of the solvents could fully dissolve the varnish, but they did induce different degrees of swelling. The swelling was measured using the percentage of mass increase of the varnish fragment after immersion according to the following formula ((mass before immersion- mass before immersion) ÷ mass before immersion × 100%); see Supplementary Materials: Table S1. Tol induced the highest swelling followed by EA. MEK, MEK/1-PeOH, and EA/PC showed less swelling but satisfactory results. The varnish fragments immersed in PC exhibited a minimum swelling, while those immersed in EtOH and WS showed almost no changes.

#### 3.4.2. Hydrogel Swelling Tests on the Collected Varnish Fragments

Based on the results obtained from the solubility/swelling tests, ethanol and white spirit were excluded from the follow-up investigations because of their low efficiencies. Since the swelling effects of both EA and MEK were to some extent comparable to that of Tol, it was decided to exclude Tol in view of its hazardous effects on human health [51].

Consequently, the test set of organic solvents/solvent mixtures were narrowed down to five solutions; these were loaded into the PVA–B/AG hydrogel in low amounts to decrease their environmental and health impacts. The hydrogel was composed of 3% PVA, 1% agarose, and 0.6% borax, in addition to the selected organic solvent(s), namely EA, PC, EA/PC, MEK, and MEK/1-PeOH. The preparation process was carried out as described elsewhere [26,52].

Two different concentrations of each solvent/solvent mixture were tested. The hydrogel composites were applied to the varnish fragments for 30 min, and the swelling of the varnish was evaluated on the basis of the mass increase. The best results were obtained with higher concentrations of solvents/solvent mixtures; the highest degree of swelling was obtained with the mixture of 5%/5% MEK/1-PeOH (see Supplementary Materials: Table S1). The swelling results were slightly different by immersion, which may be attributed to the difference in the concentration of the solvents loaded into the hydrogel and to the time of exposure (i.e., 5 min for the immersion test and 30 min for the hydrogel test).

### 3.5. Gel Cleaning Tests on the Wall Painting

Cleaning tests on the wall painting were conducted for two main reasons. The first reason was to evaluate the efficiency of the selected solvents loaded into the PVA–B/AG hydrogel in removing the varnish from the painting surface. The second reason was to test the sensitivity of different paint layers to the aforementioned hydrogel composites. For these reasons, the tests were executed over three stages. In the first stage, five hydrogel composites were applied to small spots using different contact times. In the second phase, the composites that gave the best results in the first stage were tested out on larger spots. The third stage involved testing the same hydrogel composites on sensitive paint layers in order to choose the most effective one. The results were evaluated depending on visual examinations under normal light, raking light, and UV light, in addition to ATR–FTIR spectroscopy.

#### 3.5.1. First Stage

First, the better hydrogel composite(s) for removing the varnish layer without damaging the paint layer underneath were identified, as well as the optimal contact time.

**Figure 5:**
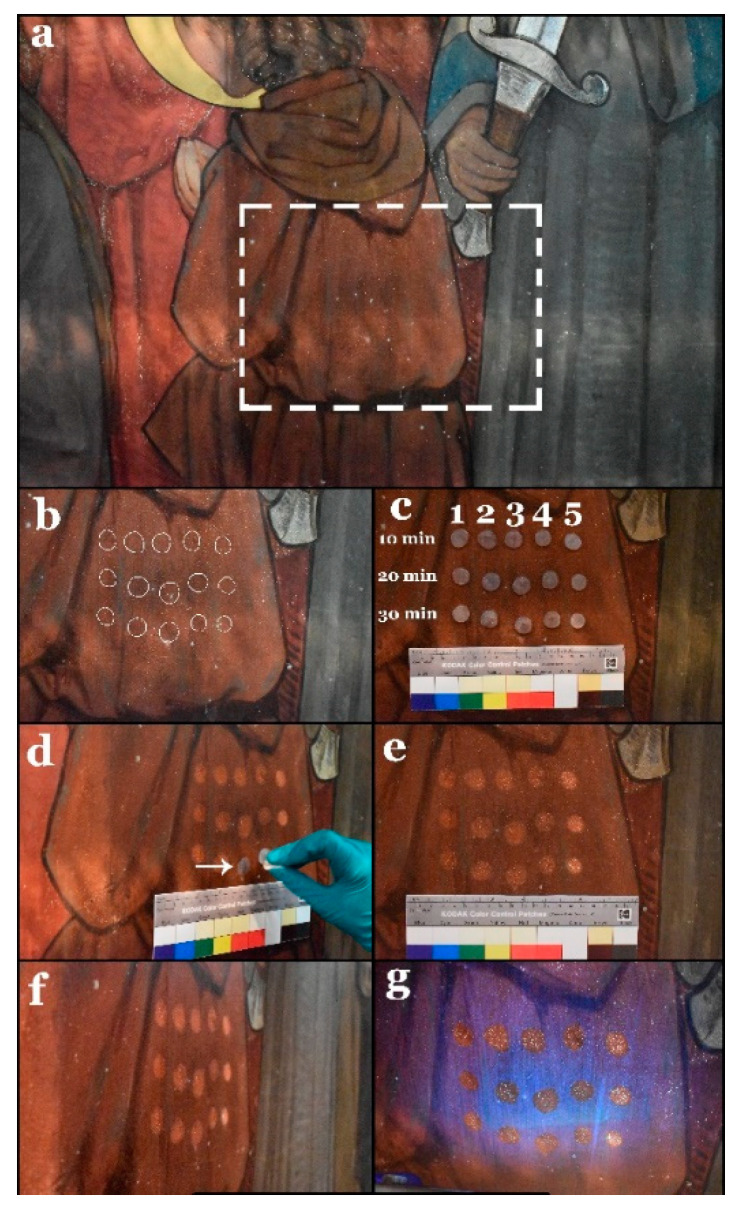
(**a**) General view of the selected area for the first stage tests. (**b**) Selected spots before the treatment indicated by white circles. (**c**) The five hydrogel composites applied to the surface during three different contact times: 10, 20, and 30 min. The numbers 1 to 5 represent the hydrogels loaded with 10% EA, 10% PC, 5%/5% EA/PC, 10% MEK, and 5%/5% MEK/1-PeOH, respectively. (**d**) Treatment of the surface with a dry cotton ball after peeling off the hydrogel pellets. The arrow refers to the swelling of the varnish resulting in a whitish haze. (**e**) View of the selected area after gel cleaning treatments. (**f**) View of the same area under raking light. (**g**) The same view under UV light showing the disappearance of the varnish fluorescence and the severe damage that occurred at the treated spots with the 5%/5% MEK/1-PeOH hydrogel composite (right-most column of test spots).

To achieve this, the five hydrogel composites were applied in small hydrogel pellets (with a diameter of *c.* 1.5 cm and a *c.* 0.3 cm thickness) on the wall painting for 10, 20, and 30 min (see Figure 5a–c). After the hydrogel composites were peeled off, the varnish layer was noticeably whitish because of the swelling effect; the pretreated varnish could be rapidly and gently rubbed away by means of a dry cotton ball (see Figure 5d). Visually, the hydrogel composite, loaded with 5/5% MEK/1-PeOH, induced severe damage to the paint layer (see Figure 5e,f). The results of 10% EA, 10% PC, and 5/5% EA/PC hydrogel composites were more or less similar. On the other hand, the 10% MEK hydrogel composite yielded unsatisfactory results: varnish removal was difficult, possibly leaving varnish residues. Regarding the contact times, there were no significant differences among them; thus, during the following test activities, only the shortest exposure time was employed.

The cleaning efficiency was evaluated by ATR–FTIR spectroscopy. Spectra were collected from the areas treated with the different hydrogel composites and from an untreated varnished area with the same color/paint. For better evaluation, an unvarnished spot on the same color/paint layer was also analyzed to represent the state of the original surface.

**Figure 6:**
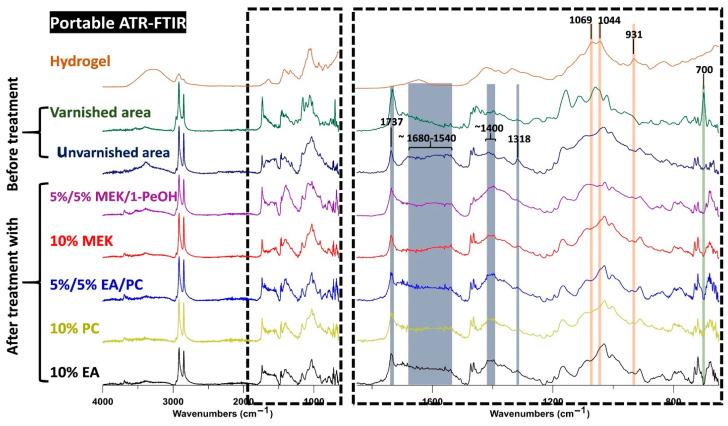
ATR–FTIR spectra of hydrogel, unvarnished and varnished paint layers, and spots treated with the five tested hydrogels in the first-stage tests.

The original painting had bands in the range of 1540 to 1680 cm^−1^, which were probably linked to the presence of carboxylates (see Figure 6). The intensity of these bands was comparable to the intensity of the band at 1737 cm^−1^ linked to the use of drying oil as a binding medium. In the spectra of the spot cleaned with MEK, this proportion was different (the band at 1737 cm^−1^ is higher), probably because of the presence of varnish remains, which have a band at 1726 cm^−1^ contributing to the enhanced intensity of the band at 1737 cm^−1^. Additionally, the painting layer showed a band at 1318 cm^−1^, which most likely corresponds to oxalates resulting from the degradation of the organic compounds and thus was a band that originated from the painting layer. This band was much less intense in the spectra of the region cleaned with MEK, which can be explained by the presence of varnish remains on the surface that partially covered the painting layer. This result is in agreement with the visual examinations.

In the spot treated with 5%/5% MEK/1-PeOH hydrogel composite, some changes were observed around 1400 cm^−1^. In this spectral range, the unvarnished and the varnished painting had several bands that were probably linked to CH_3_ and CH_2_ bending. However, in the spectra of the region cleaned with 5%/5% MEK/1-PeOH hydrogel composite, this region showed an intense band corresponding to lead carbonate (1416 cm^−1^), which was also found in the priming coat. This suggests a partial removal of the painting layer, thereby exposing the preparation so that the associated compounds showed more intense bands. Furthermore, the bands related to carboxylates (bands between 1680 and 1540 cm^−1^) were lower in intensity, which is consistent with the partial removal of the paint layer.

Finally, the spectra of the spots cleaned with 10% EA, 10% PC, and 5%/5% EA/PC hydrogel composites showed no clear differences compared with those of the unvarnished areas. To confirm these conclusions and select the most suitable hydrogel composites, more tests were performed.

The collected spectra of the treated spots also provided information about the potential presence of hydrogel residues. It is relevant to note that the characteristic intense bands of the hydrogel (at 1069, 1044, and 931 cm^−1^) were absent in all of the spectra of the treated spots; this indicates that the hydrogel left no (ATR–FTIR-detectable) residues on the surface.

#### 3.5.2. Second Stage

**Figure 7:**
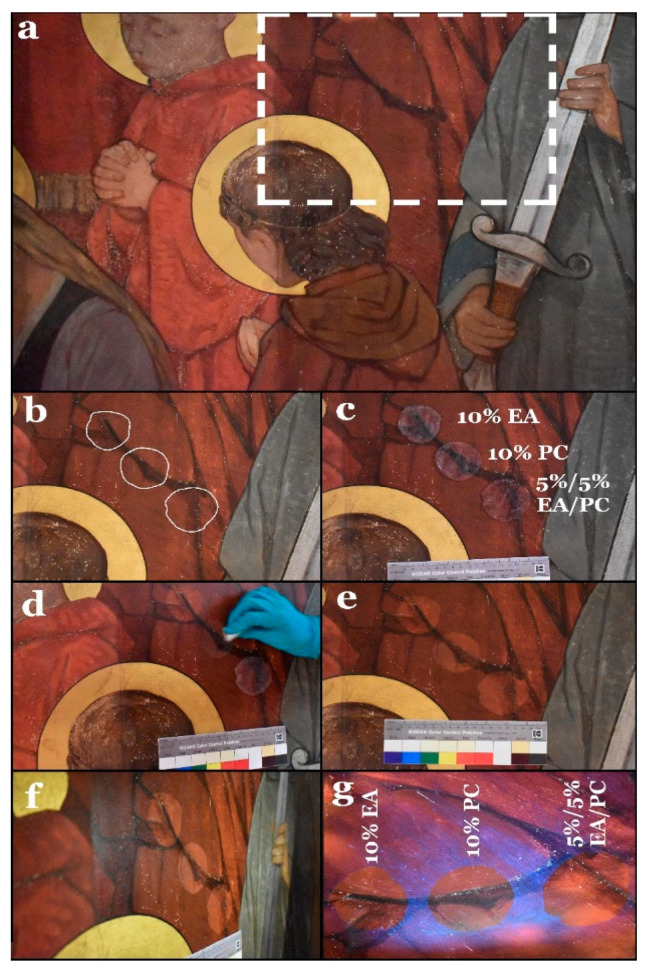
(**a**) General view of the selected area for the second-stage tests. (**b**) Selected areas before gel treatments. (**c**) The selected three hydrogel composites during application. (**d**) Removal of the swelled varnish after peeling off the hydrogel using a dry cotton ball. (**e**) View of the surface after the treatment. (**f**) The same view under raking light. (**g**) The same view under UV light.

Given the good performance demonstrated by the hydrogels loaded with 10% EA, 10% PC, and 5%/5% EA/PC, these gels were applied to larger areas (with a diameter of *c.* 5 cm and *c.* 0.3 cm thickness) of the same color/paint layer in order to verify the results obtained in the first test (see Figure 7a–d). The gel application time was 10 min.

The effects of the three hydrogel composites were verified by examination of the treated areas under visible and UV light. As in the first stage, there were no differences between the results of the three hydrogel composites (Figure 7e–g); therefore, it was decided to test them on an area of the wall painting featuring a more sensitive paint layer in order to obtain measurable differences in the response.

#### 3.5.3. Third Stage

**Figure 8:**
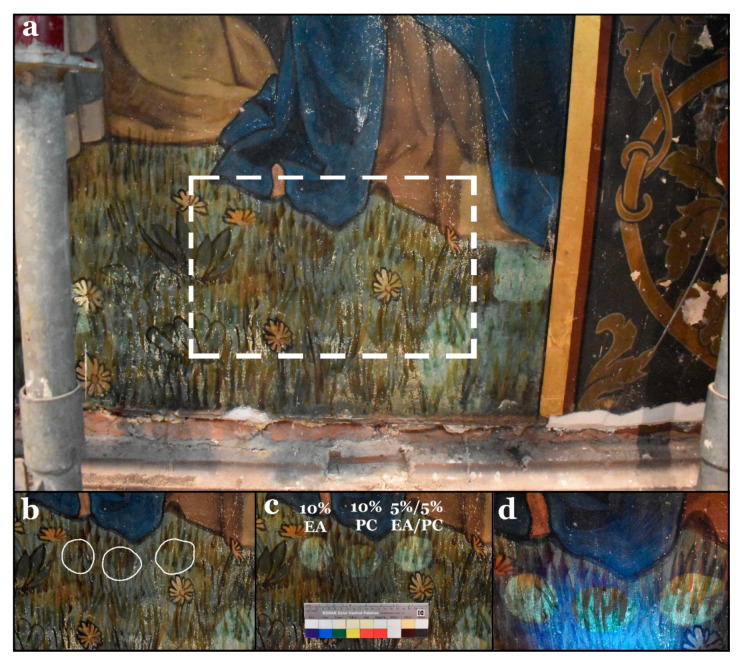
(**a**) General view of the selected area for the third-stage tests. (**b**) Selected areas before treatment. (**c**) The surface after treatment with the three hydrogel composites. Spots treated with the 10% EA and 5%/5% EA/PC hydrogel composites were overcleaned. (**d**) The same view under UV light showing the same observation regarding the overcleaning effect.

The green/brown areas of the wall painting (representing grass) are composed of superimposed paint layers (see Figure 8a). As can be seen in Supplementary Figure S3b (results of previous varnish removal tests using traditional methods), these areas proved to be very sensitive to any mechanical action. In this regard, the 10% EA, 10% PC, and 5/5% EA/PC hydrogel composites were tested following the same cleaning criteria adopted in the second-stage test. According to the visual examinations, the 10% PC hydrogel composite showed better results than the other two. Both 10% EA and 5%/5% EA/PC hydrogel composites gave rise to a slight overcleaning of the paint layer, which can be explained as a partial removal of the paint layer and the appearance of the white priming coat underneath (Figure 8b–d).

**Figure 9:**
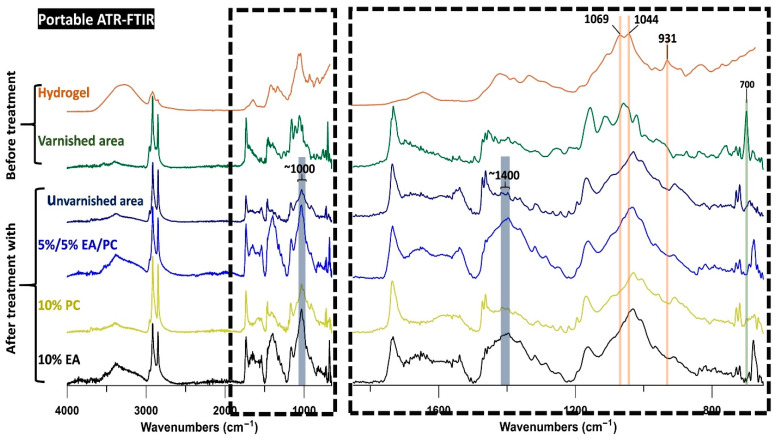
ATR–FTIR spectra of hydrogel, unvarnished and varnished areas, and the spots treated with three hydrogel composites (5%/5% EA/PC, 10% PC, and 10% EA) in the third-stage tests.

The ATR–FTIR measurements agree with the conclusions obtained from visual examination (see Figure 9). Indeed, spots cleaned with 10% EA and 5%/5% EA/PC showed a more intense band at ~1400 cm^−1^ related to the lead white present in the priming coat. This suggests that the paint layer was partially removed during cleaning. Additionally, the spot treated with the 10% PC hydrogel composite exhibited more intense bands around 2900 cm^−1^ that were more intense than the ones assigned to the pigments (around 1000 cm^−1^); this was likely related to a higher proportion of the binding medium (long CH_2_ chains). In the spots cleaned with 10% EA and 5%/5% EA/PC hydrogel composites, the same bands were less intense, indicating that the cleaning process partially removed the binding medium. In general, the spectra of the unvarnished area were very similar to the spectra of the areas cleaned with the 10% PC hydrogel composite, consistent with a small alteration of the painting layer after cleaning.

Similar to the results obtained from the first test, the hydrogel did not leave ATR–FTIR-detectable residues on the treated spots.

From all the results mentioned above, it can be concluded that the hydrogel loaded with 10% PC provided the best cleaning results overall, especially when treating areas with sensitive paint layers.

### 3.6. Implementation of Gel Cleaning Treatment of the Wall Painting and Verification of Varnish Removal

When applying the hydrogel on a large scale, it was also possible to make use of its self-healing characteristic by applying several smaller hydrogel slabs (5 × 5 cm^2^) next to each other on the wall and letting them merge. After the treatment, the hydrogel was cut and peeled off piece by piece, followed by the removal of the swollen varnish with a dry cotton ball. During the application of the hydrogel on larger areas, the concentrations of the PVA–B/AG hydrogel were slightly adjusted to 3%/1.5% PVA–B/AG for two reasons. First, the adjustment increased the liquid retention of the hydrogel, thus facilitating the reuse of the hydrogel for more than one application. Because of the low porosity of the wall painting surface, it was possible to reuse the same hydrogel patch approximately three times in some cases before it darkened because of the absorption of the varnish by the hydrogel Second, the adjustment improved the free-shaping of the hydrogel for selective applications and enhanced both of its shape-stability and workability [52]. This minor change in the concentration had no effect on the efficiency of the treatment, as it was noticed in the first-stage tests that different application times did not result in a significant difference.

**Figure 10:**
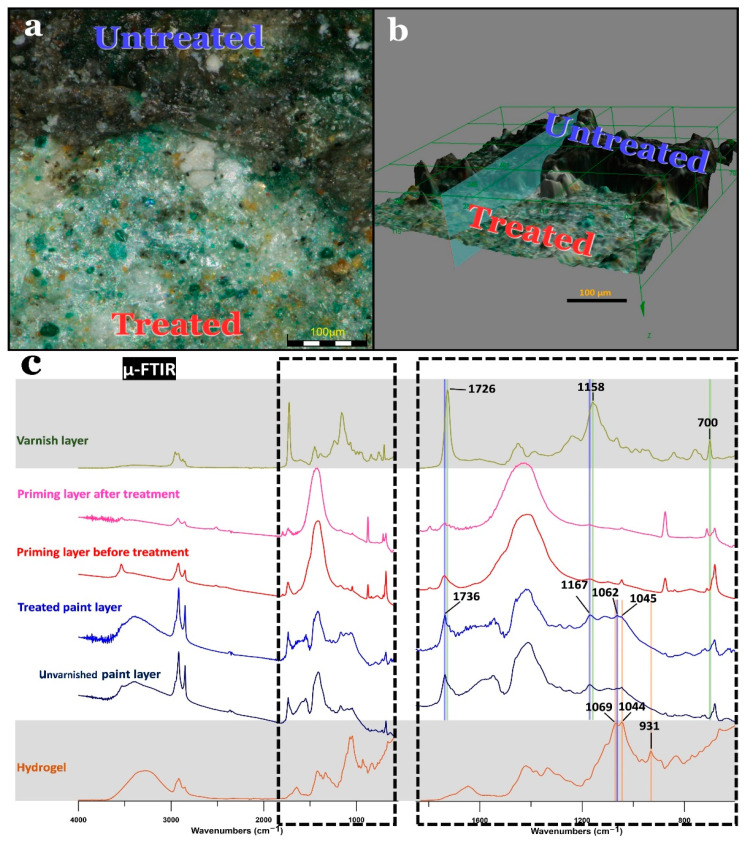
(**a**) Photomicrograph showing the boundary between a treated and an untreated area of a green area of the wall painting. The untreated area shows the darkened varnish layer, while the treated area illustrates the total removal of the varnish layer, revealing the original color of the surface. (**b**) 3D photomicrograph of the same view revealing the presence of a thick, darkened varnish layer. (**c**) µ-FTIR spectra of the varnish, priming coat before and after the treatment, treated and nontreated paint layers, and hydrogel.

To study the removal of the varnish, a paint sample at the boundary between a gel-treated and an untreated green area was collected and subjected to a number of measurements (Figure 10). Examination of the sample under the three-dimensional microscope showed the darkened varnish layer obscuring the green paint layer in the untreated area. On the other hand, the treated area of the sample showed that the varnish was completely removed and revealed the original appearance of the paint layer, with no hydrogel residues visible (Figure 10a,b).

In order to assess the effects of the gel treatment on the binding medium, fragments from treated and untreated paint areas were collected and analyzed by µ-FTIR spectroscopy. It was noticeable that the spectra of the unvarnished and treated paint layers were very similar (Figure 10c). This indicates that the solvent used for varnish removal caused no changes detected by FTIR. With respect to varnish removal efficiency, the most intense bands of the varnish at 1726 and 1158 cm^−1^ were in a similar position to the bands at 1736 and 1167 cm^−1^ of the drying oil (Figure 10c). An increase in these bands after the cleaning could be attributed to the infiltration of the varnish. There was an additional less intense peak of the styrene component of the varnish at 700 cm^−1^ that did not overlap with any bands from the paint, allowing it to be used as corroboration. As can be seen, after the cleaning there was no increase in the bands related to the varnish. This means that the varnish was successfully removed from the paint layer; if any varnish remained, it was in an amount low enough not to be detected by µ-FTIR. Furthermore, fragments from the priming coat were measured to investigate the infiltration of the varnish into the substrate after gel cleaning. Similar to the paint layer, there was no increase in the bands related to the varnish itself; this confirms that the varnish did not diffuse into the bulk of the paint strata during or after the treatment.

Regarding hydrogel residues investigations, the band at 931 cm^−1^ was only present in the hydrogel and can be used to corroborate the results. Both the paint layer and hydrogel contain a double band around 1050 cm^−1^ (Figure 10c). Specifically, the gel showed two peaks at 1044 and 1069 cm^−1^, while in the paint layer they appeared at 1045 and 1062 cm^−1^. These peaks were more intense in the hydrogel, so an increase in these bands after the cleaning would be linked to the remains of hydrogel on the surface. However, the band at 931 cm^−1^ was not present in the painting after the cleaning. In addition, no increase could be seen in the bands at 1069 and 1044 cm^−1^ after the cleaning. The slight shift of the band at 1062 cm^−1^ from the painting compared with the band at 1069 cm^−1^ from the hydrogel could still be seen in the spectra after the treatment, proving that the band at 1069 cm^−1^ had no significant increase (Supplementary Figure S6). Thus, it can be concluded that there were no µ-FTIR-detectable remains of the hydrogel. Finally, the best cleaning method, i.e. the gel composite loaded with 10% PC, was used to remove the varnish layer on a larger scale.

### 3.7. Final Results

**Figure 11:**
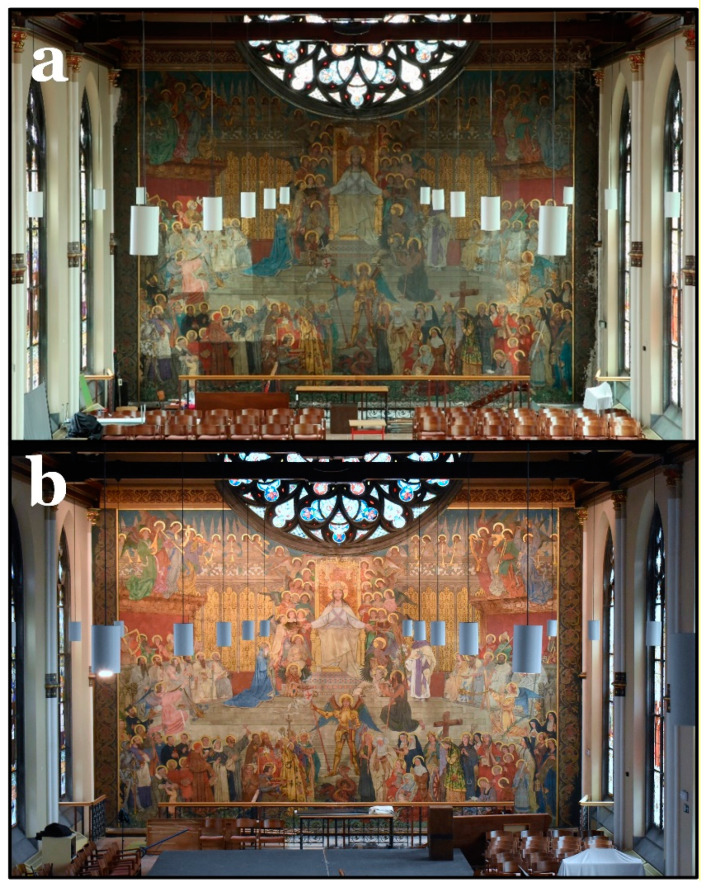
(**a**) The wall painting during the initial steps of conservation. (**b**) Final results of the wall painting after conservation and removal of the dark varnish layer.

The final results of the conservation of the wall painting are presented in Figure 11. It can be seen that the dark veil caused by the deteriorated varnish could be completely removed, revealing the original colors of the wall painting. The gilded parts now show a shiny appearance again that was previously masked by the varnish. On the other hand, in the non-gilded areas, the original overall matt appearance of the wall painting could be restored.

## 4. Conclusions

A 19th-century neo-Gothic wall painting at Sint-Jan Berchmanscollege, located in Antwerp, Belgium, was coated with a varnish layer in a previous conservation treatment; the varnish was identified as a poly(*n*-butyl acrylate-methyl methacrylate/styrene) terpolymer. This layer has undergone severe deterioration, which resulted in a strong dark-brown appearance, obscuring the paint layers and representations underneath.

Several mechanical and chemical cleaning methods were previously adopted in an attempt to remove this obscuring layer; they all failed to eliminate it without damaging the underlying paint layers. Therefore, gel cleaning was proposed as an alternative, more controllable method of swelling and dissolving the varnish. A PVA–B/AG double network hydrogel was selected as a gelling material. Various swelling tests with various solvents/solvent mixtures were systematically performed in the laboratory on varnish fragments collected from the wall painting. Then, the solvents/solvent mixtures with good swelling effects, namely EA, PC, EA/PC, MEK, and MEK/1-PeOH, were incorporated into the hydrogel and tested to remove the varnish from small areas of the wall painting itself. The results were evaluated in situ by employing a multi-technique approach (visible light, UV light, and portable FTIR spectroscopy). The obtained data showed that the hydrogel composite loaded with 10% PC was the most efficient and safe cleaning method for the varnish removal on a larger scale. The efficiency of the treatment was also examined with OM and µ-FTIR spectroscopy, allowing the verification of (a) the lack of alteration in the paint layer after the gel treatment, (b) the successful varnish removal, (c) the absence of infiltration of the varnish into the preparation layers, and (d) the lack of hydrogel residues on the treated surface. The application of the gel-based varnish removal strategy was applied to the entire ‘Last Judgment’ wall painting, in which the original colors and textures were very satisfactorily restored.

In conclusion, the gel cleaning method can provide a solution to some challenging wall painting cleaning treatments, such as varnish removal from sensitive paint layers. Moreover, it can be applied on a large scale and not only on small objects.

## Supplementary Materials

The following are available online at https://www.mdpi.com/article/10.3390/polym13162651/s1, Figure S1: Sketch illustrates the stratigraphy of the wall painting. It consists of the wall of red bricks (support) and a ground layer divided into a coarse ‘arriccio’ layer (1) and a finer ‘intonaco’ layer (2). A priming coat was applied on top of the ground layer that consisted of four sublayers. Figure S2: (a) Optical microscopy image showing the arriccio layer characterized by its coarse structure. The arrows refer to organic matter, possibly straw. The green dashed circles indicate the green earth particles. (b) Microscopic image of the organic fillers used in the ground layer (i.e., straw and hair). (c) Backscattered SEM image of the same field indicated by the red rectangle in ‘a’. (d) XRD diffractogram of the arriccio layer showing the main compounds, which was based on an average of a line scan performed on ‘a’. (e) Elemental maps acquired by SEM–EDX of the same field present in ‘d’. Figure S3: (a) Top: results obtained after mechanical varnish removal with a scalpel. The treatment did not fully remove the varnish and damaged the paint layer. Bottom: results of hot air treatment, showing the removal of the varnish layer but also some damage to the paint layer. (b) Results of a number of cotton swab tests and the damage resulting from mechanical swabbing action. (c) Application of poultices with five contact times (from left to right: 10, 30, 60, 90, and 120 min). (d) Photomicrograph illustrating the formation of a whitish haze and cellulose residues on the surface after poultice treatment. Figure S4: Part of solvents/solvent mixtures tested in both previous cleaning attempts and solubility/swelling tests. Figure S5: (a) ATR–FTIR spectrum of the varnish layer. (b) Peeled-off varnish from the wall painting illustrating the darkening/browning of the varnish. Figure S6: µ-FTIR spectra show the characteristic bands of the hydrogel and their absence from the treated paint layer, proving the absence of hydrogel residues after the treatment. Table S1: Evaluation of swelling and cleaning tests performed in the laboratory on the varnish fragments. In addition to the sections: 1. Previous cleaning attempts and 2. Varnish identification.

## References

[1] Petersen K., May E., Jones M. (2006). Wall Paintings: Aspects of Deterioration and Restoration. Conservation Science: Heritage Materials.

[2] Horie V. (2010). Materials for Conservation: Organic Consolidants, Adhesives and Coatings.

[3] Mora P., Mora L., Philippot P. (1984). Conservation of Wall Paintings.

[4] Mazzeo R., Sciutto G., Bonacini I., Prati S., Walsh M.J.K. (2017). Scientific Examinations of the Armenian Church Wall Paintings in Famagusta. The Armenian Church of Famagusta and the Complexity of Cypriot Heritage: Prayers Long Silent.

[5] Carretti E., Dei L. (2004). Physicochemical characterization of acrylic polymeric resins coating porous materials of artistic interest. Prog. Org. Coat..

[6] Stulik D., Dorge V., Miller D., Khanjian H., Institute G.C., Carlson J., Khandekar N., Wolbers R., Petersen W.C. (2004). Solvent Gels for the Cleaning of Works of Art: The Residue Question.

[7] Michalski S. (1990). A physical model of the cleaning of oil paint. Stud. Conserv..

[8] Baglioni M., Jáidar Benavides Y., Desprat-Drapela A., Giorgi R. (2015). Amphiphile-based nanofludis for the removal of styrene/acrylate coatings: Cleaning of stucco decoration in the Uaxactun archeological site (Guatemala). J. Cult. Herit..

[9] Baglioni M., Jàidar Benavides Y., Berti D., Giorgi R., Keiderling U., Baglioni P. (2015). An amine-oxide surfactant-based microemulsion for the cleaning of works of art. J. Colloid Interface Sci..

[10] Guizzo S., Tortolini C., Pepi F., Leonelli F., Mazzei F., Di Turo F., Favero G. (2019). Application of microemulsions for the removal of synthetic resins from paintings on canvas. Nat. Prod. Res..

[11] Andreotti A., Brown W.P., Camaiti M., Colombini M.P., DeCruz A. (2016). Diagnosis of materials and effectiveness of Er:YAG laser cleaning as complementary treatment in a panel painting attributed to Lluís Borrassà (fifteenth century). Appl. Phys. A.

[12] Brunetto A., Bono G., Frezzato F. (2020). Er:YAG laser cleaning of ‘San Marziale in Gloria’ by Jacopo Tintoretto in the Church of San Marziale, Venice. J. Inst. Conserv..

[13] Pouli P., Paun I.-A., Bounos G., Georgiou S., Fotakis C. (2008). The potential of UV femtosecond laser ablation for varnish removal in the restoration of painted works of art. Appl. Surf. Sci..

[14] Samorì C., Galletti P., Giorgini L., Mazzeo R., Mazzocchetti L., Prati S., Sciutto G., Volpi F., Tagliavini E. (2016). The Green Attitude in Art Conservation: Polyhydroxybutyrate–based Gels for the Cleaning of Oil Paintings. ChemistrySelect.

[15] Carretti E., Dei L., Weiss R.G., Baglioni P. (2008). A new class of gels for the conservation of painted surfaces. J. Cult. Herit..

[16] Angelova L.V., Ormsby B., Townsend J., Wolbers R. (2017). Gels in the Conservation of Art.

[17] Carretti E., Grassi S., Cossalter M., Natali I., Caminati G., Weiss R.G., Baglioni P., Dei L. (2009). Poly(vinyl alcohol)−Borate Hydro/Cosolvent Gels: Viscoelastic Properties, Solubilizing Power, and Application to Art Conservation. Langmuir.

[18] Carretti E., Natali I., Matarrese C., Bracco P., Weiss R.G., Baglioni P., Salvini A., Dei L. (2010). A new family of high viscosity polymeric dispersions for cleaning easel paintings. J. Cult. Herit..

[19] Lazidou D., Teknetzi I., Karapanagiotis I., Ritzoulis C., Panayiotou C. (2019). Poly(vinyl alcohol)-borax films as cleaning agents for icons. Archaeol. Anthropol. Sci..

[20] Duncan T.T., Berrie B.H., Weiss R.G. (2017). Soft, Peelable Organogels from Partially Hydrolyzed Poly(vinyl acetate) and Benzene-1,4-diboronic Acid: Applications to Clean Works of Art. ACS Appl. Mater. Interfaces.

[21] Pensabene Buemi L., Petruzzellis M.L., Chelazzi D., Baglioni M., Mastrangelo R., Giorgi R., Baglioni P. (2020). Twin-chain polymer networks loaded with nanostructured fluids for the selective removal of a non-original varnish from Picasso’s “L’Atelier” at the Peggy Guggenheim Collection, Venice. Herit. Sci..

[22] Moskalik-Detalle A., Assoun J., Joseph F., Martiny M.-L., Monfort M., Angelova L.V., Ormsby B., Townsend J., Wolbers R. (2017). Conservation of murals by Eugène Delacroix at Saint Sulpice, Paris. Gels in the Conservation of Art.

[23] Varadinova-Papadaki S., Angelova L.V., Ormsby B., Townsend J., Wolbers R. (2017). Gels for removing varnish and surface stains from Bulgarian icons. Gels in the Conservation of Art.

[24] Miguirditchian M., Engel N., Desvois L., Capra A.-L., Angelova L.V., Ormsby B., Townsend J., Wolbers R. (2017). Cleaning the Adolphe Roger murals at the Church of Notre Dame de Lorette, Paris. Gels in the Conservation of Art.

[25] Al-Emam E., Motawea A.G., Caen J., Janssens K. (2021). Soot removal from ancient Egyptian complex painted surfaces using a double network gel: Empirical tests on the ceiling of the sanctuary of Osiris in the temple of Seti I—Abydos. Herit. Sci..

[26] Al-Emam E., Motawea A.G., Janssens K., Caen J. (2019). Evaluation of polyvinyl alcohol–borax/agarose (PVA–B/AG) blend hydrogels for removal of deteriorated consolidants from ancient Egyptian wall paintings. Herit. Sci..

[27] Carson T., Cerrito J. (2003). New Catholic Encyclopedia. 2, 2.

[28] Tosf H., Church C. (2016). Manual of the St. John Berchmans Sanctuary Society: With a Sketch of the Saint’s Life.

[29] Prims F. (1949). Op de Gronden van Sint-Jan-Berchmans College: Van Houtmere tot Carmelkloster, stapelhuis en Gymnasium.

[30] Kunsten K.M.v.S., Buyck J. (1977). Catalogus Schilderijen 19de en 20ste Eeuw.

[31] Nauts H. (1988). Catalogus Stedelijk Kunstbezit Sint-Niklaas.

[32] Dotremont G., Martiny V.G. (1971). De Stichting Godecharle, 1871–1971.

[33] Coenjaerts P., Winckelmans P. (1989). Sint-Jan Berchmans: Een Eeuw Collegeleven.

[34] Yang X., Ji X., Cao Y., Yu T. (2019). Studies on wall painting materials and techniques at two historic buildings in Gyantse, Tibet. Herit. Sci..

[35] Schmidt B.A., Ziemann M.A., Pentzien S., Gabsch T., Koch W., Krüger J. (2016). Technical analysis of a Central Asian wall painting detached from a Buddhist cave temple on the northern Silk Road. Stud. Conserv..

[36] de la Rie E.R. (1982). Fluorescence of paint and varnish layers (Part 1). Stud. Conserv..

[37] de Viguerie L., Payard P.A., Portero E., Walter P., Cotte M. (2016). The drying of linseed oil investigated by Fourier transform infrared spectroscopy: Historical recipes and influence of lead compounds. Prog. Org. Coat..

[38] Gabrieli F., Rosi F., Vichi A., Cartechini L., Pensabene Buemi L., Kazarian S.G., Miliani C. (2017). Revealing the Nature and Distribution of Metal Carboxylates in Jackson Pollock’s Alchemy (1947) by Micro-Attenuated Total Reflection FT-IR Spectroscopic Imaging. Anal. Chem..

[39] Beltran V., Salvadó N., Butí S., Cinque G. (2015). Micro infrared spectroscopy discrimination capability of compounds in complex matrices of thin layers in real sample coatings from artworks. Microchem. J..

[40] Siidra O., Nekrasova D., Depmeier W., Chukanov N., Zaitsev A., Turner R. (2018). Hydrocerussite-related minerals and materials: Structural principles, chemical variations and infrared spectroscopy. Acta Crystallogr. Sect. B.

[41] Legodi M.A., de Waal D., Potgieter J.H., Potgieter S.S. (2001). Rapid determination of CaCO_3_ in mixtures utilising FT—IR spectroscopy. Miner. Eng..

[42] Cather S., Howard H. (1986). The use of wax and wax-resin preservatives on English mediaeval wall paintings: Rationale and consequences. Stud. Conserv..

[43] Beckett B., Brajer I. (2013). The search for an enduring painting technique: Franz Fernbach and his encaustic technique as a restoration procedure for wall-paintings in the early nineteenth century. Conservation in the Nineteenth Century.

[44] Ford T., Rizzo A., Hendriks E., Frøysaker T., Caruso F. (2019). A non-invasive screening study of varnishes applied to three paintings by Edvard Munch using portable diffuse reflectance infrared Fourier transform spectroscopy (DRIFTS). Herit. Sci..

[45] Azémard C., Vieillescazes C., Ménager M. (2014). Effect of photodegradation on the identification of natural varnishes by FT-IR spectroscopy. Microchem. J..

[46] The Infrared and Raman Users Group (IRUG). http://www.irug.org/.

[47] Learner T. (2004). Analysis of Modern Paints.

[48] Miller E., Rainer L., Rivera A.B. (2004). The Nebamun Wall Paintings of the British Museum. The Conservation of Decorated Surfaces on Earthen Architecture: Proceedings from the International Colloquium Organized by the Getty Conservation Institute and the National Park Service Mesa Verde National Park, Colorado, USA 22–25 September 2004.

[49] Bandaranayake S., Agnew N. (1997). The Dambulla Rock Temple Complex, Sri Lanka: Ten years of management, research, and conservation. Conservation of Ancient Sites on the Silk Road: Proceedings of an International Conference on the Conservation of Grotto Sites, Mogao Grottoes, Dunhuang, China, 3–8 October 1993.

[50] Phenix A., Wolbers R., Stoner J.H., Rushfield R. (2012). Removal of varnish: Organic solvents as cleaning agents. The Conservation of Easel Paintings.

[51] Alder C.M., Hayler J.D., Henderson R.K., Redman A.M., Shukla L., Shuster L.E., Sneddon H.F. (2016). Updating and further expanding GSK’s solvent sustainability guide. Green Chem..

[52] Al-Emam E., Soenen H., Caen J., Janssens K. (2020). Characterization of polyvinyl alcohol-borax/agarose (PVA-B/AG) double network hydrogel utilized for the cleaning of works of art. Herit. Sci..

