# Supplementary material for: Removal of a Past Varnish Treatment from a 19th-Century Belgian Wall Painting by Means of a Solvent-Loaded Double Network Hydrogel
Source: Polymers (Basel). 2021 Aug 10;13(16):2651. doi: 10.3390/polym13162651 (PMC8399542; doi:10.3390/polym13162651)
Supplement: Supplementary file 1 [file polymers-13-02651-s001.zip › polymers-1323279-SM.pdf]

Removal of a Past Varnish Treatment from a 19th-Century Belgian Wall Painting by Means of a Solvent-Loaded Double Network Hydrogel

Ehab Al-Emam [1,2,*], Victoria Beltran [2], Steven De Meyer [2], Gert Nuyts [2], Vera Wetemans [3], Karolien De Wael [2], Joost Caen [3] and Koen Janssens [2]

[1] Department of Conservation, Faculty of Archaeology, Sohag University, 82524, Sohag, Egypt

[2] AXES, Faculty of Science, University of Antwerp, Groenenborgerlaan 171, 2020 Antwerp, Belgium; victoria.beltran@uantwerpen.be (V.B.); steven.demeyer@uantwerpen.be (S.D.M.); gert.nuyts@uantwerpen.be (G.N.); karolien.dewael@uantwerpen.be (K.D.W.); koen.janssens@uantwerpen.be (K.J.)

[3] ARCHES, Faculty of Design Sciences, University of Antwerp, Mutsaardstraat 31, 2000 Antwerp, Belgium; wetemansvera@gmail.com (V.W.); joost.caen@uantwerpen.be (J.C.)

* Correspondence: ehab_alemam@arch.sohag.edu.eg or ehab.alemam@uantwerpen.be

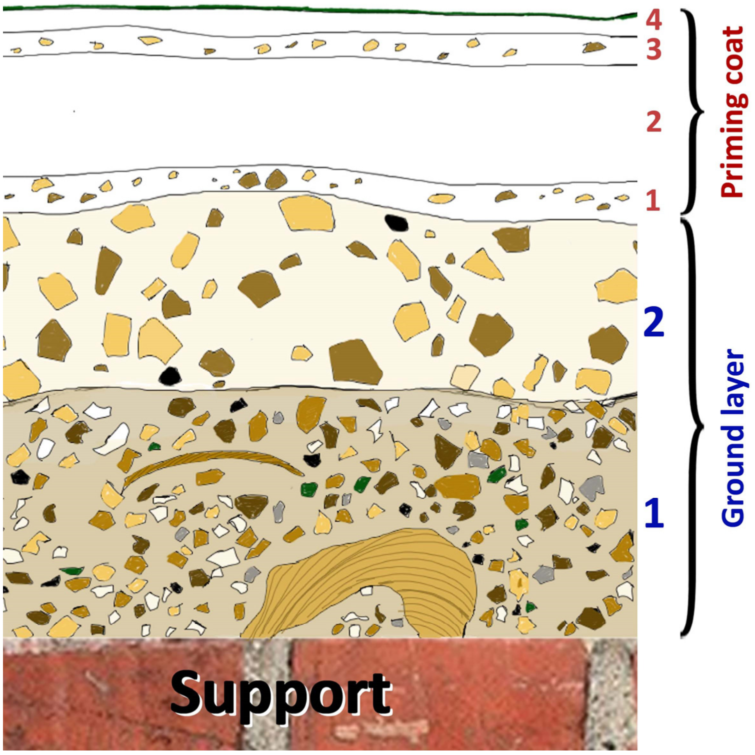

Figure S1: Sketch illustrates the stratigraphy of the wall painting. It consists of the wall of red bricks (support) and a ground layer divided into a coarse 'arriccio' layer (1) and a finer 'intonaco' layer (2). A priming coat was applied on top of the ground layer that consisted of four sublayers.

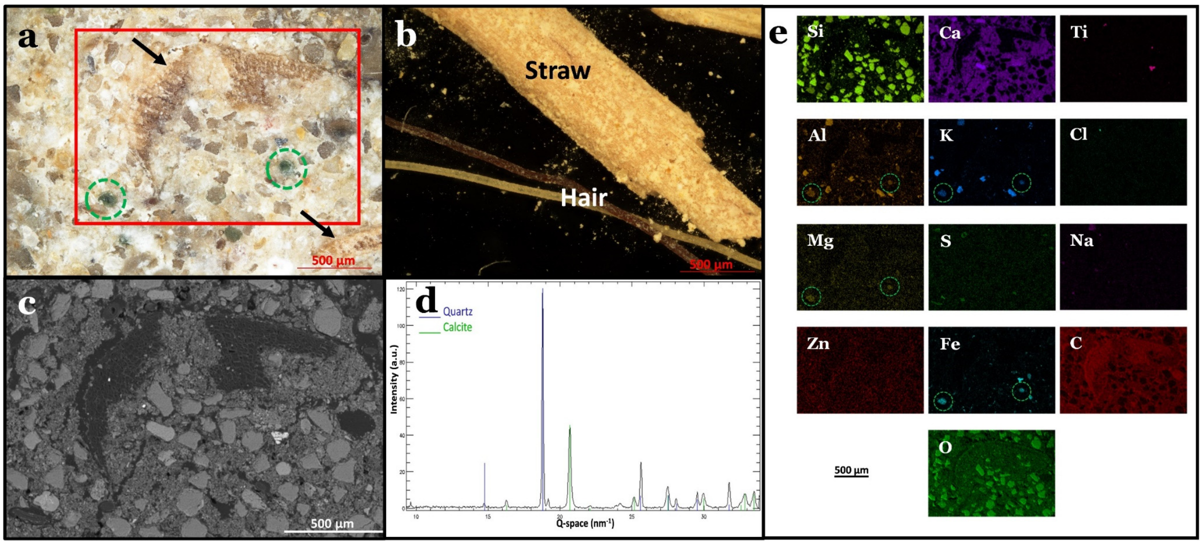

Figure S2: a) Optical microscopy image showing the arriccio layer characterized by its coarse structure. The arrows refer to organic matter, possibly straw. The green dashed circles indicates the green earth particles. b) Microscopic image of the organic fillers used in the ground layer (i.e., straw and hair). c) Backscattered SEM image of the same field indicated by the red rectangle in 'a'. d) XRD diffractogram of the arriccio layer showing the main compounds; which was based on an average of a linescan performed on 'a'. e) Elemental maps acquired by SEM–EDX of the same field present in 'd'.

1. Previous cleaning attempts

A conservation campaign started in 2017 by the conservation-restoration department, University of Antwerp. In order to remove of the varnish, a number of cleaning attempts were conducted on the unidentified varnish. These attempts were based on testing several traditional techniques and they can be summarized as follows:

1.1. Mechanical cleaning tests

Scalpel tests: The varnish layer was removed mechanically by means of a scalpel. However, it was not possible to fully remove it, moreover this method caused severe damage to the paint layer underneath.

Hot air tests: Hot air flow of a heat gun was exploited in order to soften the varnish layer, afterwards dry cotton balls were rubbed on the surface to remove this layer. This technique managed to remove the varnish layer with minimal negative effect on the paint layer compared to the scalpel cleaning tests (as indicated in Figure S3a).

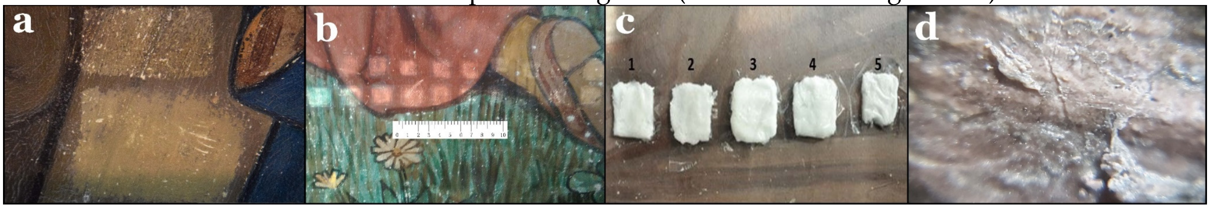

Figure S3: a) Top: results obtained after mechanical varnish removal with a scalpel. The treatment did not fully remove the varnish and damaged the paint layer. Bottom: results of hot air treatment, showing the removal of the varnish layer but also some damage to the paint layer. b) Results of a number of cotton swab tests and the damage resulting from mechanical swabbing action. c) Application of poultices with five contact times (from left to right: 10, 30, 60, 90, and 120 minutes). d) Photomicrograph illustrating the formation of a whitish haze and cellulose residues on the surface after poultice treatment.

1.2. Chemical cleaning tests

Cotton swab cleaning tests: These tests were based on a set of 24 solvent mixtures composed variable amounts of *AC*, *EtOH*, *EA*, *Tol*, cyclohexane and, water. These mixtures are described in [1] and they occupy a wide range spot on the Teas diagram which facilitates identifying the most efficient one to dissolve this specific varnish.

The cleaning tests were carried out by dipping a cotton swab into the solvent mixtures and applying it on the surface of the wall painting. Although the swab cleaning technique was aggressive and caused detachment to the paint layers (see Figure S3b), a number of solvent mixtures were able to dissolve the varnish with minimal effect on the paint surface. Those solvent mixtures were: AC/EtOH (75%/25%) and EtOH/water (50%/50%), which have been plotted on the Teas diagram in Figure S4.

Poultice cleaning test: In order to avoid the damage observed in the swab cleaning to increase the liquid retention of the solvents, the blend of EtOH and water (50%/50%) was chosen and loaded into a poultice to remove the varnish. The poultice was prepared by mixing 2% Tylose (methyl hydroxyethyl cellulose) and the aforementioned solvent mixture. Later, paper pulp was added until the desired consistency was acquired. This poultice was applied to the wall painting via five different contact times (10, 30, 60, 90, 120 minutes) and then covered with Melinex film to hinder the evaporation of the solvents (see Figure S3c). The poultices did

not completely remove the varnish layer, furthermore they left behind a whitish haze and paper pulp residues on the treated spots; as indicated in Figure S3d.

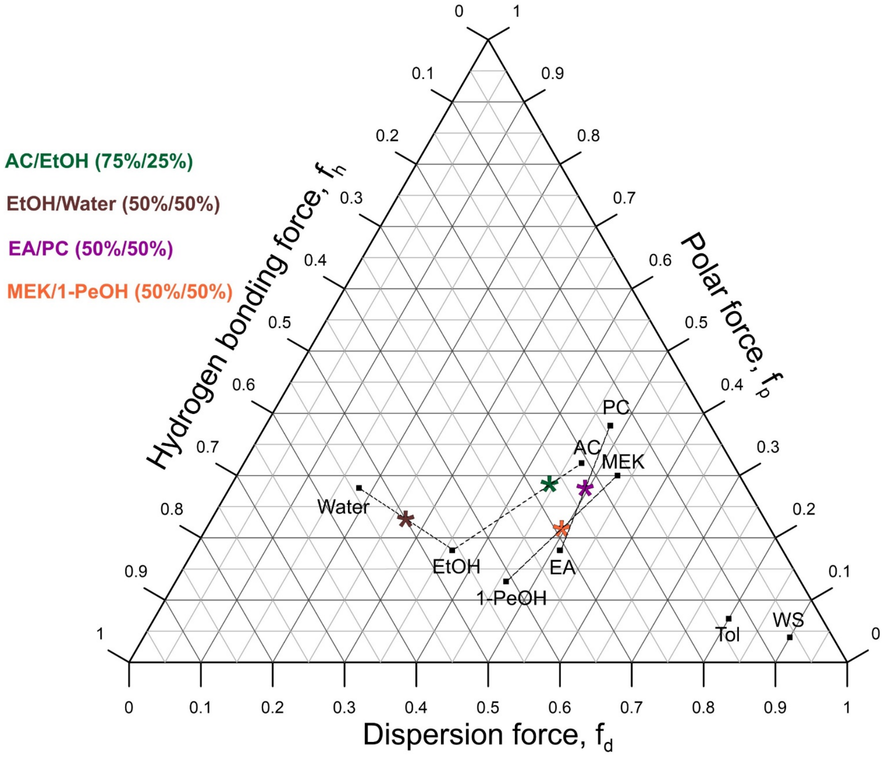

Figure S4: Part of solvents/solvent mixtures tested in both previous cleaning attempts and solubility/swelling tests.

In conclusion, all previously described cleaning methods lead to damaging the paint layer especially the green/brown areas (imitating the grass) which proved to be sensitive to the tested methods. Moreover, poultice cleaning led to partial removal of the varnish in addition to leaving residues on the treated surface. Therefore, gel cleaning was tested due to their good retention of solvents and being less aggressive to the paint layers.

2. Varnish identification

The ATR–FTIR spectrum of the varnish shows the presence of CH_2 bands at 2959 and 2873 cm^{-1} (asymmetrical and symmetrical C-H stretching of the CH_3) (see Figure S5a), at 2919 and 2849 cm^{-1} (asymmetrical and symmetrical C-H stretching of the CH_2), at 1451 and 1385 cm^{-1} (C-H bending) and at 757 cm^{-1} and 701 cm^{-1} (C-H rocking) [2,3]. There are also carbonyl groups at 1726 cm^{-1} (C=O stretching). The medium intensity peak at 1238, the strong broad peak at 1158, and the peak at 1029 cm^{-1} are indicative of stretching bands of C-O and C-C [2,4,5]. The peak at 990 cm^{-1} represents the rocking vibration of CH_3 group which is characteristic of methyl methacrylate while the double bands at 963 and 942 cm^{-1} in addition to the weak-to-medium band at 841 cm^{-1} refer to the butyl acrylate units [6]. At higher wavenumbers, three weak vibrations appear at 3084, 3061, and 3026 cm^{-1} of the aromatic C-H stretching, consistent with the presence of styrene. Furthermore, the weak peak at 1602 cm^{-1} with a shoulder at 1583 cm^{-1} and the two peaks at 1492 cm^{-1} and 1451 cm^{-1} are due to the aromatic ring breathing [2,3].

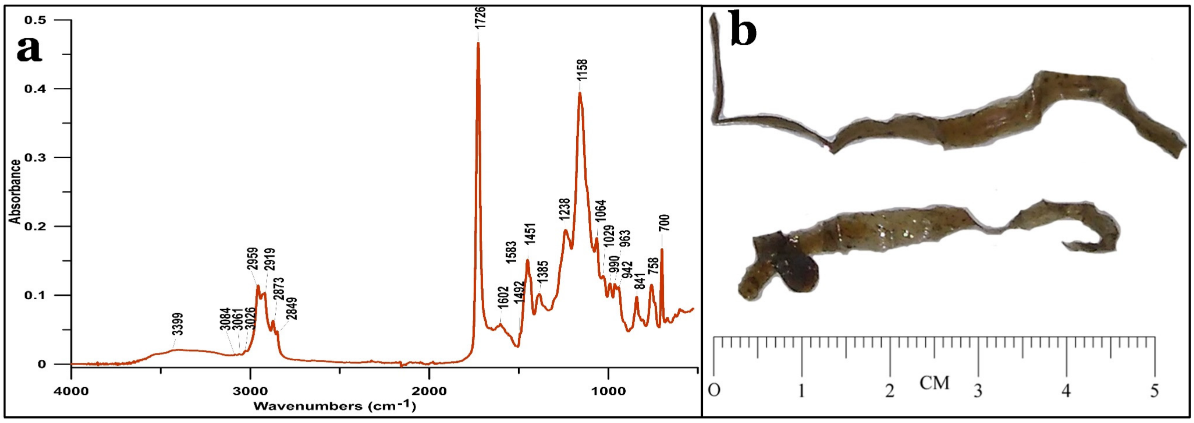

Figure S5: a) ATR–FTIR spectrum of the varnish layer. b) Peeled-off varnish from the wall painting illustrating the darkening/browning of the varnish.

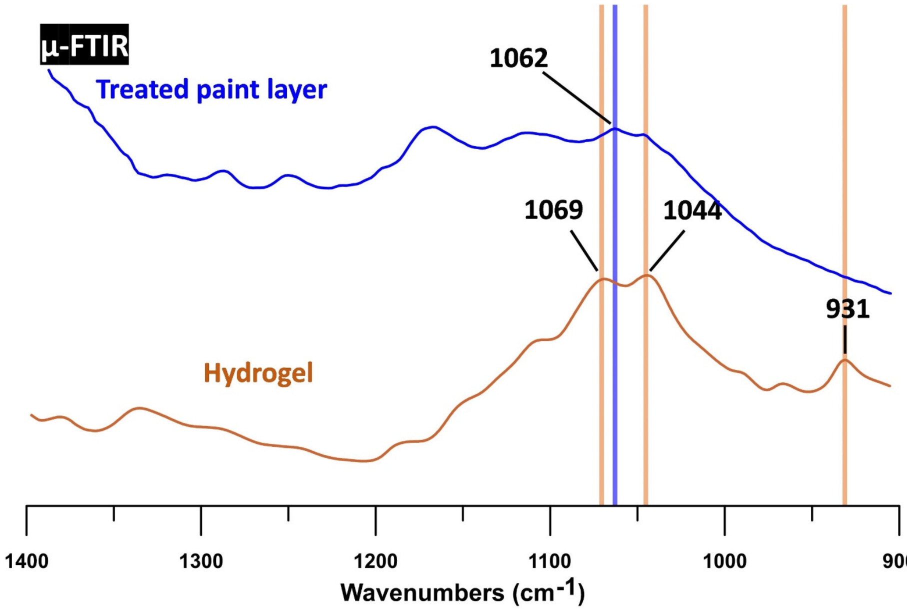

Figure S6: μ–FTIR spectra show the characteristic bands of the hydrogel and their absence from the treated paint layer, proving the absence of hydrogel residues after the treatment.

Table S1: Evaluation of swelling and cleaning tests performed in the laboratory on the varnish fragments

	Swelling tests on the varnish fragments		Gel swelling tests on the varnish fragments	
	Concentration	Efficacy (weight increase %)	Concentration	Efficacy (weight increase %)
EA	100%	622 ±49	10%	5.7 ±0.5
PC	100%	85 ±5	10%	3.4 ±0.9
EtOH	100%	14 ±7	Not tested	Not tested
WS	100%	16 ±6	Not tested	Not tested
Tol	100%	718 ±131	Not tested	Not tested
EA/PC	50%/50%	316 ±44	5%/5%	4.6 ±1
MEK	100%	551 ±26	10%	4.6 ±1.3
MEK/1-PeOH	50%/50%	483 ±55	5%/5%	11.7 ±2.6

References

1. Pietsch, A. *Lösemittel: Ein Leitfaden für die restauratorische Praxis*; Theiss: Stuttgart, Germany, 2005.
2. Learner, T. *Analysis of modern paints*; The Getty Conservation Institute: Los Angeles, CA, USA, 2004.
3. Chatzi, E.G.; Kammona, O.; Kentepozidou, A.; Kiparissides, C. Infrared spectra and compositional analysis of styrene/2-ethylhexyl acrylate copolymers. *Macromolecular Chemistry and Physics* **1997**, *198*, 2409-2420, doi:10.1002/macp.1997.021980805.
4. Chércoles Asensio, R.; San Andrés Moya, M.; de la Roja, J.M.; Gómez, M. Analytical characterization of polymers used in conservation and restoration by ATR-FTIR spectroscopy. *Analytical and Bioanalytical Chemistry* **2009**, *395*, 2081-2096, doi:10.1007/s00216-009-3201-2.
5. Chiantore, O.; Lazzari, M. Characterization of Acrylic Resins. *International Journal of Polymer Analysis and Characterization* **1996**, *2*, 395-408, doi:10.1080/10236669608033358.
6. Scalarone, D.; Chiantore, O. Separation techniques for the analysis of artists' acrylic emulsion paints. *Journal of Separation Science* **2004**, *27*, 263-274, doi:10.1002/jssc.200301638.
